# Serum vitamin D Levels in patients with medication-related osteonecrosis of the jaw: a systematic review and meta-analysis

**DOI:** 10.1186/s12903-026-08303-9

**Published:** 2026-04-14

**Authors:** Alireza Daneshvar, Kimia Daneshvar, Farhan Musaie, Sobhan Dashti, Niloofar Talebi Yazdi, Reza Tabrizi

**Affiliations:** 1 Dental Research Center, Research Institute of Dental Sciences, Shahid Beheshti University of Medical Sciences, Danshjoo BLVD, Velenjak, Shahid Chamran Highway, 1983963113, Tehran, Iran; 2 Ophthalmic Research Center, Research Institute for Ophthalmology and Vision Science, Shahid Beheshti University of Medical Sciences, Tehran, Iran; 3 Dentistry student, Dental Branch, Islamic Azad University, Tehran, Iran; 4 Department of Oral and Maxillofacial Surgery, School of Dentistry, Shahid Beheshti University of Medical Sciences, Tehran, Iran

**Keywords:** Bisphosphonate-associated osteonecrosis of the jaw, MRONJ, Bisphosphonates, Antiresorptive agents, Vitamin D, Meta-analysis

## Abstract

**Objectives:**

To compare serum 25-hydroxyvitamin D [25(OH)D] levels between patients with medication-related osteonecrosis of the jaw (MRONJ) and antiresorptive-exposed controls.

**Materials and methods:**

Following PRISMA guidelines, PubMed, Scopus, Web of Science, and Embase were searched through September 2025 for studies including adults (≥ 18 years) treated with bisphosphonates or denosumab that reported 25(OH)D levels in MRONJ cases and exposed controls. Random-effects models generated standardized mean differences (SMDs) with 95% confidence intervals (CIs). Heterogeneity, influence and subgroup analyses, random-effects meta-regression, and publication bias (Egger’s and Begg’s tests) were assessed.

**Results:**

Six studies (five case–control, one cohort; 184 MRONJ, 175 controls) met inclusion criteria. Mean serum 25(OH)D was 33.16 ± 4.27 ng/mL in MRONJ patients and 36.86 ± 5.02 ng/mL in controls. The SMD was − 0.06 (95% CI, − 0.51 to 0.39; *p* = 0.79) with heterogeneity (I² = 75.0%; *p* < 0.001). After exclusion of two influential studies, heterogeneity decreased to 0% and the SMD remained non-significant (− 0.11; 95% CI, − 0.36 to 0.15; *p* = 0.41). Subgroup analyses by indication/population (mixed osteoporosis plus oncologic versus exclusively oncologic) showed no significant differences. Meta-regression indicated that control sample size and bisphosphonate exposure duration in MRONJ groups were associated with effect size. The certainty of evidence for the analyzed outcome was very low.

**Conclusions:**

Postdiagnosis serum 25(OH)D levels appear similar in MRONJ patients and antiresorptive-exposed controls; however, the evidence is limited and of very low certainty, so conclusions should be interpreted cautiously.

**Clinical relevance:**

In antiresorptive-treated adults, serum 25-hydroxyvitamin D levels measured after diagnosis do not differ meaningfully between patients with MRONJ and exposed controls, suggesting vitamin D is unlikely to be a primary determinant of MRONJ occurrence but may still modify disease course.

**Trial registration:**

CRD420251172147.

**Supplementary Information:**

The online version contains supplementary material available at 10.1186/s12903-026-08303-9.

## Introduction

Medication-related osteonecrosis of the jaw (MRONJ) is a rare but potentially debilitating adverse effect associated primarily with antiresorptive therapies (e.g., bisphosphonates and denosumab) and, less commonly, certain antiangiogenic/targeted agents [1]. Current evidence indicates that MRONJ arises from a multifactorial interplay between pharmacologically suppressed osteoclastic bone turnover in the jaws, compromised mucosal repair and barrier function with superimposed infection/inflammation, and alterations in host immune and vascular/angiogenic pathways; these processes may be triggered or exacerbated by dentoalveolar procedures and/or chronic oral inflammatory conditions [2]. Clinically, the AAOMS case definition requires current or prior exposure to antiresorptive therapy (alone or in combination with immune modulators and/or antiangiogenic medications), the presence of exposed bone or bone that can be probed through an intraoral or extraoral fistula in the maxillofacial region persisting for more than 8 weeks, and no history of radiotherapy to the jaws or metastatic disease involving the jaws [1]. MRONJ can present with pain, swelling, infection, loosening of teeth, and radiographic changes, and advanced disease may lead to complications such as extraoral fistulae and pathologic fracture, substantially impairing oral function and quality of life [3]. The incidence of MRONJ is increasing, particularly with the widespread use of these medications for bone-related diseases such as osteoporosis and cancer [4].

Vitamin D, a secosteroid hormone, is crucial for calcium absorption and bone mineralization and is positively associated with bone mineral density. Vitamin D deficiency reduces calcium absorption, prompting the release of calcium from bone to maintain normal blood levels and thereby weakening bone structure through ongoing turnover and resorption [5, 6]. Additionally, vitamin D influences bone remodeling through VDR signaling in osteoblast-lineage cells. This signaling modulates the RANKL/OPG axis and osteoclastogenesis [7]. Vitamin D deficiency may exacerbate the adverse skeletal effects of long-term bisphosphonate therapy, potentially contributing to the pathogenesis of MRONJ [8].

Although several studies suggest a potential link between vitamin D deficiency and MRONJ development, the evidence remains inconsistent. Some research has found significantly lower serum 25-hydroxyvitamin D [25(OH)D] levels in MRONJ patients [9, 10], while other studies have failed to establish a clear association [11]. The role of vitamin D in the development of MRONJ remains debated, with inconsistent findings across populations and study designs. Prior studies are heterogeneous, and quantitative syntheses focusing specifically on serum 25(OH)D in MRONJ remain limited. This meta-analysis aims to evaluate differences in serum 25(OH)D levels between individuals with MRONJ and antiresorptive-exposed controls without MRONJ. Our goal is to clarify the potential association between vitamin D deficiency and MRONJ development.

## Materials and methods

This systematic review and meta-analysis was conducted in accordance with the PRISMA (Preferred Reporting Items for Systematic Reviews and Meta-Analyses) guidelines [12] and the Institute of Medicine’s standards for systematic reviews [13]. The study protocol was prospectively registered in the International Prospective Register of Systematic Reviews (PROSPERO; registration number CRD420251172147).

### Eligibility criteria

The study followed the population, exposure, comparison, and outcome (PECO) guidelines as follows: In adults (≥ 18 years) with prior exposure to antiresorptive therapies (bisphosphonates and/or denosumab) for any clinical indication (Population, P), this study evaluated whether the presence of medication-related osteonecrosis of the jaw (MRONJ), diagnosed using explicit clinical and/or radiographic criteria (Exposure, E), compared with antiresorptive-exposed individuals without MRONJ (Comparison, C), is associated with differences in serum 25-hydroxyvitamin D [25(OH)D] levels (Outcome, O).

To be eligible for quantitative synthesis, studies had to include both MRONJ cases and an antiresorptive-exposed non-MRONJ comparator group and report extractable serum 25(OH)D data for each group. We excluded studies without a comparator group (e.g., case series), studies with irrelevant populations (non-adults, non-antiresorptive-exposed participants), irrelevant outcomes (no serum 25(OH)D), unclear MRONJ definition, non-original publications (reviews, editorials), conference abstracts/protocols, non-human studies, duplicate datasets, and reports with insufficient or non-extractable data.

### Information sources and search strategy

A comprehensive literature search was conducted independently by two reviewers (FM and AD) in PubMed, Scopus, Web of Science, and Embase, with each database searched from inception to 24 September 2025 (a consistent end date across databases). Search strategies were developed using a logical Boolean combination (OR/AND) of core concepts: medication-related osteonecrosis of the jaw (e.g., MRONJ/ONJ/BRONJ/ARONJ; osteonecrosis/necrosis AND jaw/mandible/maxilla) and relevant agents (e.g., bisphosphonates, antiresorptives, denosumab, antiangiogenics), combined with terms for vitamin D and 25-hydroxyvitamin D (e.g., vitamin D, 25(OH)D/25OHD, calcidiol/calcifediol, cholecalciferol, ergocalciferol). Full database-specific search strategies are provided in Online Resource 1. Reference lists of included studies were also screened manually to identify additional eligible publications.

### Selection process

All retrieved records were imported into EndNote 21 for duplicate removal and subsequently exported to Rayyan, an online platform for systematic review screening. Study selection was conducted in two stages by two independent reviewers (FM and AD), working independently and in duplicate, according to predefined inclusion and exclusion criteria. In the first stage, titles and abstracts were screened for relevance, followed by full-text assessment in the second stage to determine eligibility. Any discrepancies were resolved through discussion and consultation.

### Data collection process and data items

Two reviewers (AD and KD) independently performed data extraction, and any disagreements were resolved through discussion and full-text review. Data were extracted for:

- Study characteristics: first author/year, study design, and country.
- Population: indication, type of antiresorptive agent, route of administration, and MRONJ definition.
- Study groups: sample sizes of cases and controls, and matching criteria.
- Measurement variables: 25(OH)D units, sampling timepoints, and assay methods.
- Outcomes: postdiagnosis serum 25(OH)D levels (mean ± SD) for cases and controls.

When studies reported multiple post-diagnosis 25(OH)D measurements, we extracted a single prespecified time point per study (the earliest post-diagnosis assessment closest to diagnosis) for the primary meta-analysis to preserve independence; additional time points were recorded descriptively.

We extracted serum 25(OH)D as mean ± SD for cases and controls whenever reported. When SDs were not reported but individual participant (patient-level) 25(OH)D values were available in the primary publication, we calculated group means and sample SDs directly from those values. If neither mean ± SD nor patient-level data were available, we included a study in quantitative synthesis only when sufficient unweighted summary statistics were reported to permit valid conversion; otherwise, the study was not pooled and was discussed qualitatively.

### Study quality assessment

The quality of included studies was independently evaluated by two reviewers (AD and KD) using the Newcastle-Ottawa Scale (NOS; range 0–9). Discrepancies were resolved through discussion and, when necessary, consultation. Scores were categorized as ≤ 3 (low quality), 4–6 (moderate), and 7–9 (high).

Because the NOS is a study-quality tool (not a domain-based risk-of-bias instrument), we report NOS results as methodological quality and used them to inform the GRADE ‘study limitations/risk of bias’ domain.

### Statistical analysis

All analyses were conducted in Stata 17 (StataCorp, College Station, TX, USA).

Primary outcome: The primary outcome was the standardized mean difference (SMD) in serum 25(OH)D between MRONJ and control groups, with 95% confidence intervals (CIs). Pooled effects were estimated using DerSimonian–Laird random-effects models with inverse-variance weighting. For descriptive context, pooled group means (ng/mL) were also summarized.

Heterogeneity assessment: Between-study heterogeneity was quantified using Cochran’s Q (and its p value), I², and τ².

Sensitivity analyses: Influence and outlier diagnostics included leave-one-out analyses and Galbraith plots. When outliers were detected, pooled effects were re-estimated after excluding the flagged study or studies, singly and jointly, to evaluate robustness.

Subgroup analysis: Subgroups were defined by indication/population (mixed osteoporosis plus oncologic versus exclusively oncologic). Random-effects models were fit within each subgroup, and differences between subgroups were tested using a Q test for subgroup differences.

Meta-regression: Random-effects meta-regression was used to assess whether continuous moderators explained between-study variability: number of MRONJ and control participants, mean age, percent female, study latitude, bisphosphonate treatment duration, and proportion undergoing tooth extraction. Regression coefficients (β) and p values are reported.

Publication bias: Funnel plots were visually inspected; Egger’s regression test and Begg’s rank-correlation test were applied. A p value < 0.05 was considered statistically significant.

### Certainty of the meta-evidence assessment

The level of evidence was evaluated using the GRADE system [14], which classifies the certainty of evidence for meta-analysis outcomes as high, moderate, low, or very low, based on key domains including risk of bias, imprecision, inconsistency, and indirectness.

## Results

### Study selection

**Fig. 1:**
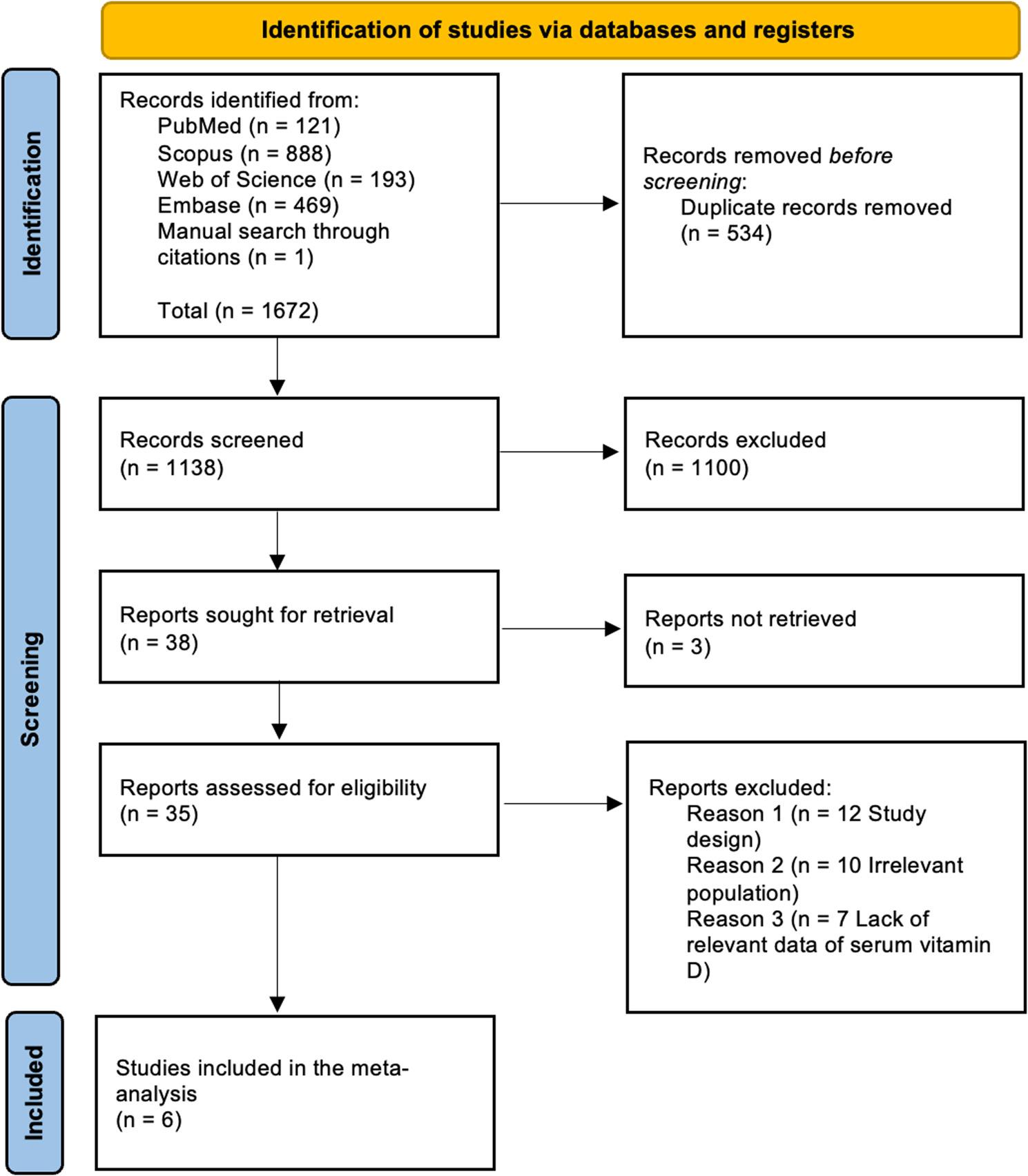
PRISMA flow diagram of literature search and selection process

The initial database search identified 1,672 records. After removing 534 duplicates, 1,138 unique records were screened by title and abstract, leading to the exclusion of 1,100 irrelevant studies. Three reports could not be retrieved. The full texts of the remaining 35 articles were assessed for eligibility, resulting in the inclusion of six studies in the meta-analysis: five case-control studies and one prospective cohort study. A flow diagram of the selection process is provided in Fig. 1.

### Study characteristics

Table 1 summarizes the key characteristics of the six studies published between 2013 and 2022. These studies were conducted across Asia, the Americas, Europe, and Oceania and included a total of 184 MRONJ cases and 175 controls. The cohorts were comparable in sex distributions (72.0% versus 74.2% female) and mean age (MRONJ: 68.3 ± 10.8 years; controls: 63.9 ± 11.4 years). Reported differences in mean vitamin D levels between MRONJ patients and controls ranged from − 34.48 to 7.00 ng/mL, with four studies reporting higher levels in the MRONJ group and two reporting lower levels. This wide variability highlights the heterogeneity across studies.

**Table 1:** Characteristics and outcomes of the included studies

Author, Year	Study design & setting	Population / indication & agent(s)	Sample size (cases, *n*)	Sample size (controls, *n*)	Measurement (unit; timing; assay)	Serum 25(OH)D (test: MRONJ/ONJ), mean ± SD	Serum 25(OH)D (control), mean ± SD
Park et al. (2022) [16]	Prospective cohort, Republic of Korea	Indication=mixed; Agent(s)=pamidronate, alendronate, ibandronate, risedronate, zoledronate; Route = IV/oral; AAOMS = 2014	24 at T1	30 at T1	Unit = ng/mL; Sampling=T0 (preoperative baseline), T1 (8-week post-op/diagnosis), and T2 (4-month post-diagnosis/follow-up); Assay=CMIA (Abbott Architect)	T0: 21.30 ± 11.28 T1: 21.33 ± 10.58 T2: 23.15 ± 11.61	T0: 19.83 ± 10.82 T1: 20.16 ± 11.22 T2: 23.62 ± 11.35
Shirani et al. (2021) [9]	Case-control, Iran	Indication = NR; Agent(s)=bisphosphonates; Route = IV/oral; AAOMS = NR	10	10	Unit = ng/mL; Sampling=post-diagnosis; Assay = NR	29.51 ± 23.723	63.99 ± 29.796
Picardo et al. (2020) [10]	Case-control, Argentina	Indication=mixed; Agent(s)=alendronate, zoledronate; Route = IV/oral; AAOMS = 2014	58	28	Unit = ng/mL; Sampling=post-diagnosis; Assay=radioimmunoassay (DiaSorin)	28.4 ± 9.4	32.5 ± 9.7
Heim et al. (2017) [17]	Case-control, Germany	Indication=mixed; Agent(s)=pamidronate, alendronate, ibandronate, risedronate, zoledronate, denosumab; Route = IV/oral/SC; AAOMS = 2014	45	18	Unit = ng/mL; Sampling=post-diagnosis; Assay = NR	20.49 ± 12.16	20.37 ± 11.32
Thumbigere-Math et al. (2016) [11]	Case-control, USA	Indication=oncology; Agent(s)=pamidronate, zoledronate; Route = IV; AAOMS = 2009	25	48	Unit = µg/L; Sampling=post-diagnosis; Assay = NR	38 ± 6	31 ± 10
Tsao et al. (2013) [15]	Case-control, Australia	Indication=oncology; Agent(s)=bisphosphonates; Route = IV; Classification=ASBMR	22	41	Unit = ng/mL; Sampling=post-diagnosis; Assay = NR	65.8 ± 23.8	65.7 ± 26.3

25(OH)D, 25-hydroxyvitamin D; AAOMS, American Association of Oral and Maxillofacial Surgeons; ASBMR, American Society for Bone and Mineral Research; CMIA, chemiluminescent microparticle immunoassay; IV, intravenous; MRONJ, medication-related osteonecrosis of the jaw; ONJ, osteonecrosis of the jaw; NR, not reported; RIA, radioimmunoassay; SC, subcutaneous; SD, standard deviation; post-op, postoperative; ng/mL, nanograms per milliliter; µg/L, micrograms per liter

Footnote

• Heim et al.: mean ± SD were calculated from patient-level 25(OH)D data reported in the article

### Study quality assessment

Methodological quality was assessed using the Newcastle-Ottawa Scale (NOS), with domain sets tailored to cohort and case-control designs (selection, comparability, and outcome/exposure). The item prompts used for each design are listed in Online Resource 2. Overall, studies demonstrated moderate methodological quality with some residual concerns. In the comparability domain, which credits adjustment for key confounders (age and sex), only the study by Tsao et al. [15] achieved two stars, indicating more comprehensive confounder control than its peers. Several studies did not receive stars in one or more selection-domain items (e.g., case definition/representativeness or control selection/definition), which contributed to lower overall NOS totals in those reports.

### Meta-analysis results

**Fig. 2:**
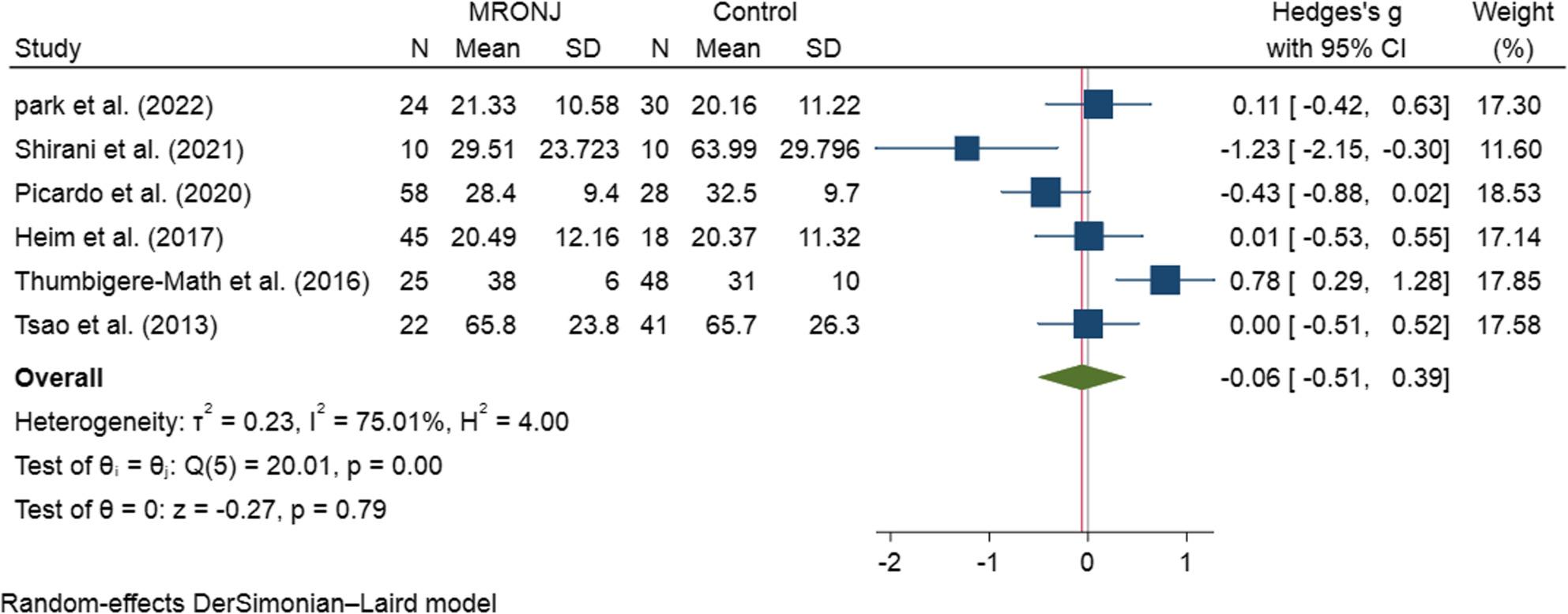
Forest plot of the standardized mean difference in serum 25(OH) vitamin D levels between MRONJ patients and controls

Mean pooled serum vitamin D was 33.16 ± 4.27 ng/mL in the MRONJ group and 36.86 ± 5.02 ng/mL in the control group. Meta-analysis revealed no statistically significant difference between the two groups (standardized mean difference [SMD], − 0.06; 95% CI, − 0.51 to 0.39; *p* = 0.79; Fig. 2). However, substantial heterogeneity was present across studies (I² = 75.0%; p for heterogeneity < 0.001), as shown in the Galbraith plot (Online Resource 3).

To explore potential sources of heterogeneity, we conducted sensitivity and subgroup analyses. The sensitivity analysis identified the studies by Shirani et al. [9] and Thumbigere-Math et al. [11] as major contributors (Online Resource 4). Excluding Shirani et al. [9] did not reduce heterogeneity or change the primary effect estimate (SMD, 0.09; 95% CI, − 0.31 to 0.49; *p* = 0.66; I² = 68.6%; p for heterogeneity = 0.01; Online Resource 5). Excluding Thumbigere-Math et al. [11] reduced heterogeneity to a moderate level while the main result remained non-significant (SMD, − 0.21; 95% CI, − 0.57 to 0.14; *p* = 0.24; I² = 51.2%; p for heterogeneity = 0.08; Online Resource 6). Excluding both studies identified as the principal contributors to heterogeneity eliminated between-study heterogeneity (I² = 0%; p for heterogeneity = 0.40) and yielded a revised pooled effect of SMD − 0.11 (95% CI, − 0.36 to 0.15; *p* = 0.41; Online Resource 7).

### Subgroup analysis

**Fig. 3:**
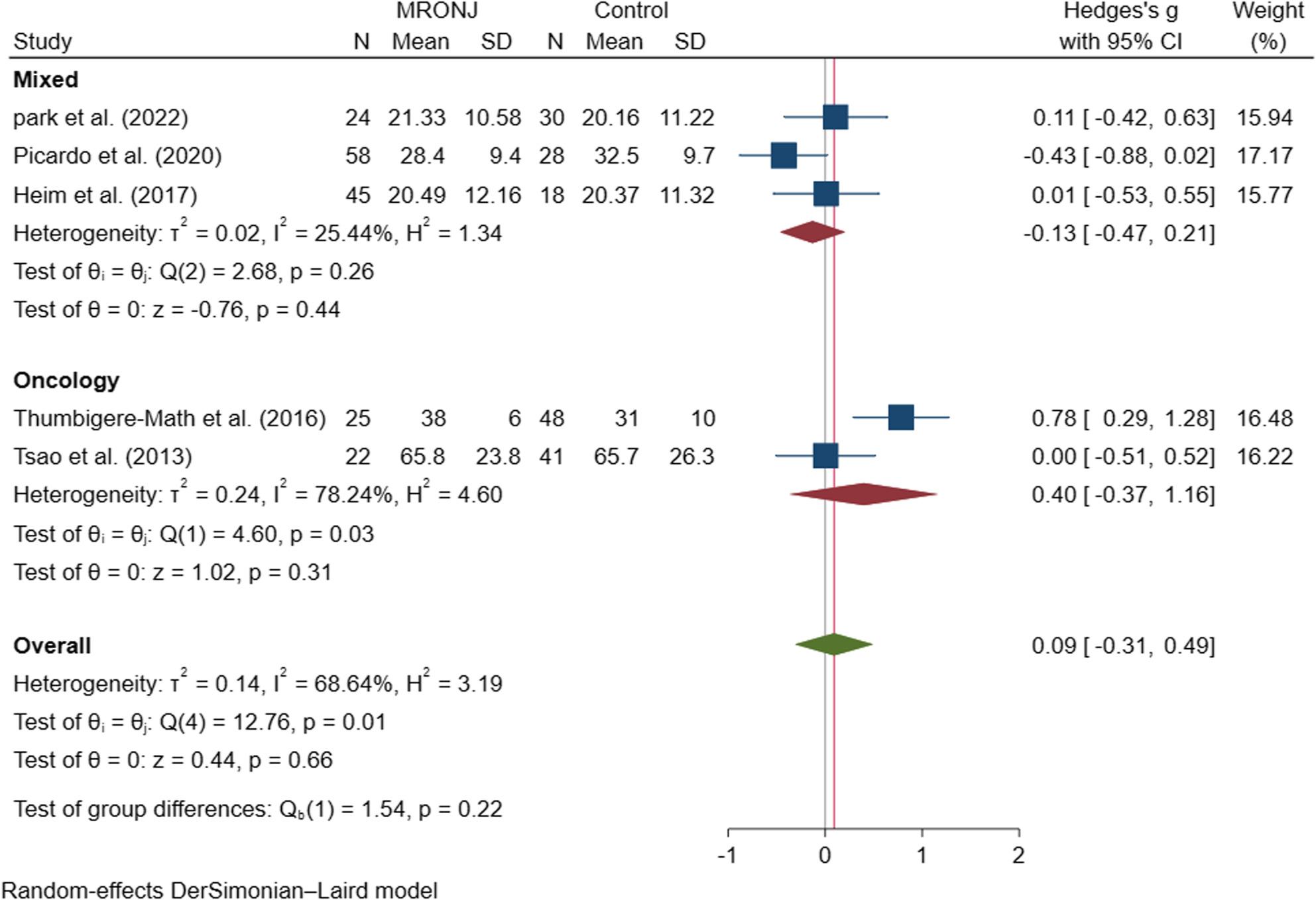
Forest plot of the standardized mean difference in serum 25(OH) vitamin D levels between MRONJ patients and controls according to the nature of population

A subgroup analysis was performed based on patient population, categorizing studies into those with mixed (oncologic plus osteoporotic) populations [10, 16, 17] and those with exclusively oncologic populations [11, 15]. In studies with mixed populations, MRONJ patients had slightly lower vitamin D levels, though the difference was not statistically significant (SMD, − 0.13; 95% CI, − 0.47 to 0.21; *p* = 0.44), with low heterogeneity (I² = 25.4%; p for heterogeneity = 0.26). In the exclusively oncologic subgroup (two studies), MRONJ patients exhibited non-significantly higher vitamin D levels (SMD, 0.40; 95% CI, − 0.37 to 1.16; *p* = 0.31), with substantial heterogeneity (I² = 78.2%; p for heterogeneity = 0.03). The test for subgroup differences indicated no statistically significant difference in vitamin D levels between population types (*p* = 0.22; Fig. 3).

### Meta-regression

The results of the meta-regression are summarized in Table 2. Two variables emerged as significant moderators of the effect size. First, the sample size of the control group showed a significant positive association with the effect size on effect estimates (β = 0.035; *p* = 0.011), indicating that studies with larger control groups tended to report slightly greater vitamin D deficiencies in the MRONJ group. Second, among clinical factors, the duration of bisphosphonate therapy in the MRONJ group was a significant moderator (β = −0.059; *p* = 0.001). This inverse relationship indicates that longer duration of bisphosphonate therapy was associated with a larger negative effect size, meaning lower vitamin D levels in patients compared to controls. In contrast, none of the other assessed variables—including mean age, percent female in either group, the geographical latitude of the study locations, or the percentage of patients undergoing tooth extraction—showed a significant association with the effect size (all *p* > 0.05).

**Table 2:** Univariable meta-regression exploring heterogeneity in SMD of serum 25(OH)D (MRONJ vs. antiresorptive-exposed controls)

Variable	B Coefficient	*p*-value	Notes
MRONJ Group Sample Size	0.0006	0.969	
Control Group Sample Size	0.0352	**0.011**	Significant positive association
Mean Age (MRONJ Group)	-0.0078	0.927	
Mean Age (Control Group)	0.0660	0.316	
% Female (MRONJ Group)	-0.4270	0.741	
% Female (Control Group)	-1.2602	0.252	
Latitude	0.0214	0.594	
BP Duration (MRONJ Group)	-0.0592	**0.001**	Significant negative association (k = 3)
BP Duration (Control Group)	0.0196	0.925	(k = 3)
Extractions (MRONJ Group)	0.6677	0.834	(k = 3)
Extractions (Control Group)	-0.3946	0.822	(k = 3)

*MRONJ* medication-related osteonecrosis of the jaw, *BP* bisphosphonate, *B* regression coefficient, *k* number of studies contributing data

Footnotes

• B indicates the change in SMD per 1-unit increase in the covariate

• Results with k < 10 are underpowered and may be unstable; interpret cautiously

Bold values indicate statistically significant results (*p* < 0.05)

### Publication bias

Despite visual asymmetry in the funnel plot (Online Resource 8), statistical analyses using Egger’s (*p* = 0.077) and Begg’s (*p* = 0.707) tests revealed no significant evidence of publication bias.

### Certainty of the evidence

Using the GRADE approach, the certainty of evidence for the primary outcome (serum 25[OH]D difference between MRONJ cases and antiresorptive-exposed controls) was rated very low (Table 3). The evidence base comprised six observational studies (184 MRONJ and 175 controls), and the pooled estimate was imprecise (SMD − 0.06; 95% CI − 0.51 to 0.39) with substantial heterogeneity (I²=75.0%). Certainty was downgraded for study limitations (informed by NOS scores, including one low-quality study and limited confounder adjustment), inconsistency (heterogeneity in the primary analysis, although reduced after removal of influential studies), and imprecision (wide confidence intervals). No statistically significant publication bias was detected using Egger’s test or Begg’s test; however, the ability to detect small-study effects was limited by the small number of included studies.

**Table 3:** GRADE certainty of evidence

GRADE domain	Judgment	Rationale
Risk of bias	Serious (− 1)	NOS scores ranged 3–6 (case–control) and 5 (cohort), including one low-quality study (score 3); comparability/adjustment was limited across studies
Inconsistency	Serious (− 1)	Substantial heterogeneity in the primary analysis (I² = 75.0%); heterogeneity decreased to 0% after excluding two influential studies, although the pooled effect remained non-significant
Indirectness	Not serious (0)	Population/exposure/comparator/outcome match the PECO question (antiresorptive-exposed adults; MRONJ vs. exposed controls; serum 25(OH)D)
Imprecision	Serious (− 1)	The CI is wide and crosses the null (SMD − 0.51 to 0.39), and total sample size is modest (*n* = 359)
Publication bias	Undetected / Unclear (0)	Funnel plot showed slight asymmetry, but Egger’s and Begg’s tests were not significant; however, power is limited with < 10 studies
Overall certainty	Very low	Observational evidence starts at low and was downgraded for risk of bias, inconsistency, and imprecision

## Discussion

The present systematic review and meta-analysis synthesizing data from six observational studies demonstrated no significant difference in serum vitamin D levels between patients with MRONJ and controls. Although the pooled effect size slightly favored lower vitamin D levels in MRONJ patients, the association did not reach statistical significance, and substantial heterogeneity was initially observed. Sensitivity analyses identified two studies as key contributors to this heterogeneity; their exclusion eliminated heterogeneity while maintaining a non-significant effect.

Our findings are consistent with Bedogni et al.; however, their results were not quantitatively pooled because 25(OH)D was reported as median (IQR) in the context of skewed/censored data and CEM-weighted Tobit modeling, which precludes reliable reconstruction of an unweighted mean ± SD for SMD-based meta-analysis. Nonetheless, they similarly found no meaningful between-group difference in vitamin D status, reporting comparable vitamin D deficiency prevalence (< 20 ng/mL) in BRONJ cases (59%) and bisphosphonate-exposed controls (62%) (95% CI − 22 to 16%; *p* = 0.77) [18].

The absence of a statistically significant difference in post-diagnosis serum 25(OH)D between MRONJ cases and antiresorptive-exposed controls should be interpreted in the context of MRONJ’s multifactorial pathogenesis and the limitations of available observational evidence. Contemporary guidance emphasizes that individual MRONJ risk cannot be reliably predicted or excluded using any single laboratory parameter; in particular, the AAOMS 2022 update notes that no biomarkers are validated for clinical decision-making and that bone-turnover markers should not be used for MRONJ risk stratification [1]. Related oncology-focused guidance similarly frames MRONJ risk assessment as clinical and exposure-based rather than laboratory-driven [19]. Within this framework, studies evaluating vitamin D status have reported mixed associations. Differences in study design, the timing of sampling relative to diagnosis or therapy changes, and the extent of confounding control likely contribute to this inconsistency. Accordingly, our pooled null estimate should be interpreted as a lack of evidence for a consistent between-group difference in post-diagnosis vitamin D status, rather than evidence of no biological role; moreover, it does not support causal inference or individual risk prediction.

Between-study heterogeneity was effectively resolved after the exclusion of influential outlier studies, Shirani et al. [9] and Thumbigere-Math et al. [11], yielding I² = 0%, which indicates a consistent pooled effect across the remaining evidence. The study by Shirani et al. [9] was a very small, unmatched case-control series (10 versus 10), with an extreme between-group separation in 25(OH)D (controls ≈ 64 ng/mL versus MRONJ ≈ 30 ng/mL) not reproduced elsewhere, and a major exposure imbalance by route (MRONJ predominantly intravenous versus controls 100% oral), all of which could plausibly confound both vitamin D status and MRONJ risk. Thumbigere-Math et al. [11] introduced directional bias through asymmetric sampling: MRONJ cases were measured after bisphosphonate discontinuation (≈ 11 months), whereas controls were sampled while on therapy, an approach that can invert expected differences and, indeed, coincided with higher 25(OH)D in cases than in controls. However, because this homogeneity was achieved by excluding two studies from a small evidence base, the pooled estimate may be sensitive to individual studies and should be interpreted cautiously.

Subgroup analyses by patient population (mixed versus exclusively oncologic) similarly revealed no statistically significant differences. However, underlying disease may confound subgroup comparisons because oncologic patients often have higher prevalence of vitamin D deficiency due to malnutrition, systemic inflammation, reduced sun exposure, and treatment-related effects, whereas osteoporosis patients may be more likely to receive routine vitamin D supplementation [20, 21]. Therefore, subgroup findings should be interpreted cautiously, particularly where the distribution of indications within mixed cohorts was not balanced or not reported.

In exploratory meta-regression, larger control sample size and longer bisphosphonate exposure duration were associated with more negative effect estimates (i.e., lower 25(OH)D levels in MRONJ cases relative to controls). The observed relationship with bisphosphonate duration may reflect factors correlated with prolonged therapy—such as greater comorbidity burden, reduced mobility and sun exposure, nutritional compromise, or concurrent medications—rather than a direct causal role of vitamin D in MRONJ development. The association with control sample size may indicate greater estimate instability in smaller studies and potential small-study effects, whereby unmeasured confounding, measurement variability, or study-specific methods exert disproportionate influence on effect estimates. Importantly, these meta-regression findings are hypothesis-generating only: with a small number of included studies, the analyses are underpowered and should be interpreted cautiously. Accordingly, they should not inform clinical decision-making or risk stratification based on vitamin D levels at this stage, but rather underscore the need for larger, well-designed prospective studies with standardized exposure definitions and pre-diagnosis vitamin D assessment.

External evidence suggests that vitamin D status may be more closely related to MRONJ severity than to its initial occurrence. In observational cohorts, lower 25(OH)D has been reported more frequently in established disease and in more advanced stages, and pre-procedural supplementation has been associated with subsequent presentation at less severe stages [22, 23].

Similarly, external studies evaluating supplementation after MRONJ diagnosis report mixed and generally modest associations with healing, with some finding no meaningful improvement in mucosal closure and others suggesting potential adverse associations in comorbid populations receiving vitamin D analogs [22, 24].

Anchored by our meta-analysis showing no significant post-diagnosis difference in serum 25(OH)D between MRONJ cases and antiresorptive-exposed controls, the evidence suggests that vitamin D is not a primary determinant of MRONJ incidence but may be a modifier of disease severity based on external evidence. This is consistent with a prophylaxis-first approach of screening and optimizing 25(OH)D before invasive dental procedures in antiresorptive-treated patients. However, once MRONJ is established, vitamin D repletion alone appears to confer limited healing benefit and is generally considered an adjunct within multimodal management.

It is important to note that all studies included in this meta-analysis measured serum vitamin D levels after MRONJ diagnosis. Consequently, the findings primarily reflect vitamin D status concurrent with established disease rather than premorbid levels. This distinction limits causal inference because disease-related factors such as changes in oral intake, systemic inflammation, or lifestyle modifications could influence serum 25(OH)D concentrations. Therefore, the non-significant differences observed between MRONJ patients and controls may underestimate any true effect of vitamin D deficiency on MRONJ risk.

In addition, the evidence base was limited, with a relatively small number of eligible studies and modest sample sizes, which reduces statistical power and increases uncertainty around the pooled estimate. Between-study heterogeneity was substantial in the primary analysis, likely reflecting differences in study populations (oncologic vs. osteoporosis indications), antiresorptive regimens (drug type, dose, and route), diagnostic criteria for MRONJ, and timing and methods of vitamin D measurement. Residual confounding is also likely, as several studies provided limited adjustment for key determinants of vitamin D status and MRONJ risk (e.g., supplementation, season, comorbidities, renal function, BMI, smoking, and concurrent medications). Finally, although small-study effects/publication bias were explored, formal assessments are underpowered with fewer than 10 studies and should be interpreted cautiously.

This meta-analysis underscores several critical knowledge gaps that warrant further investigation. First, future research should prioritize prospective, longitudinal studies designed to elucidate the relationship between pre-procedural serum vitamin D levels and the subsequent risk of developing MRONJ, which would provide a more robust basis for causal inference than the cross-sectional, postdiagnosis measurements available in the current literature. Second, there is a pressing need to evaluate the efficacy of prophylactic vitamin D supplementation in reducing MRONJ incidence in high-risk cohorts undergoing antiresorptive therapy, ideally through randomized controlled trials. Third, the role of vitamin D as a modifiable factor influencing disease progression remains poorly defined; well-designed studies are required to determine if serum 25(OH)D levels correlate with MRONJ stage and severity at diagnosis, and if adjunctive vitamin D therapy can meaningfully improve healing outcomes and reduce morbidity in established disease.

## Conclusion

In conclusion, serum 25(OH)D levels measured after MRONJ diagnosis appear similar between patients with MRONJ and antiresorptive-exposed controls. However, because the available evidence is limited and of very low certainty, this conclusion should be interpreted with caution.

## Supplementary Information

Supplementary Material 1.

Supplementary Material 2.

## Data Availability

The datasets generated and/or analysed during the current study are available from the corresponding author on reasonable request.
