# Supplementary material for: Serum vitamin D Levels in patients with medication-related osteonecrosis of the jaw: a systematic review and meta-analysis
Source: BMC Oral Health. 2026 Apr 14;26:1040. doi: 10.1186/s12903-026-08303-9 (PMC13270808; doi:10.1186/s12903-026-08303-9)
Supplement: Supplementary file 1 — Supplementary Material 1. [file 12903_2026_8303_MOESM1_ESM.pdf]

Online Resource 1. The detailed search query made to each of the databases.

Database	Search Query
PubMed	(Bisphosphonate-Associated Osteonecrosis of the Jaw[Mesh] OR mronj[tiab] OR onj[tiab] OR bronj[tiab] OR aronj[tiab] OR ((osteonecros*[tiab] OR necros*[tiab]) AND (jaw[tiab] OR jaws[tiab] OR mandib*[tiab] OR maxilla*[tiab] OR maxillofacial[tiab])) OR ((medication[tiab] OR drug*[tiab] OR bisphosphonate*[tiab] OR antiresorpt*[tiab] OR denosumab[tiab] OR antiangiogen*[tiab]) AND (osteonecros*[tiab] OR necros*[tiab]) AND (jaw[tiab] OR jaws[tiab] OR mandib*[tiab] OR maxilla*[tiab] OR maxillofacial[tiab]))) AND (Vitamin D[Mesh] OR Vitamin D Deficiency[Mesh] OR Cholecalciferol[Mesh] OR Ergocalciferols[Mesh] OR Calcifediol[Mesh] OR Hydroxycholecalciferols[Mesh] OR (vitamin[tiab] AND d[tiab]) OR 25OHD[tiab] OR (25[tiab] AND hydroxyvitamin[tiab] AND d[tiab]) OR calcidiol[tiab] OR calcifediol[tiab] OR cholecalciferol[tiab] OR ergocalciferol[tiab] OR ercalcidiol[tiab] OR hydroxycholecalciferol*[tiab])
Scopus	TITLE-ABS-KEY (mronj OR onj OR bronj OR aronj OR ((medication OR drug* OR bisphosphonate* OR antiresorpt* OR denosumab OR antiangiogen*) AND (osteonecros* OR "bone necrosis" OR necrosis) AND (jaw OR jaws OR mandib* OR maxill* OR maxillofacial)) OR "osteonecrosis of the jaw" OR "jaw osteonecrosis" OR (osteonecros* W/3 jaw)) AND TITLE-ABS-KEY ("vitamin d" OR "vitamin d status" OR "hypovitaminosis d" OR "25(OH)D" OR "25-oh-d" OR 25ohd OR "25-hydroxyvitamin d" OR "25 hydroxyvitamin d" OR calcidiol OR calcifediol OR cholecalciferol OR ergocalciferol OR "25-hydroxycholecalciferol" OR "25-hydroxyergocalciferol" OR ercalcidiol)
ISI Web of Science	TS=(mronj OR onj OR bronj OR aronj OR ((medication OR drug* OR bisphosphonate* OR antiresorpt* OR denosumab OR antiangiogen*) AND (osteonecros* OR "bone necrosis" OR necrosis) AND (jaw OR jaws OR mandib* OR maxill* OR maxillofacial)) OR "osteonecrosis of the jaw" OR "jaw osteonecrosis" OR (osteonecros* NEAR/3 jaw)) AND TS=("vitamin d" OR

	"vitamin d status" OR "hypovitaminosis d" OR "25(OH)D" OR "25-oh-d" OR 25ohd OR "25-hydroxyvitamin d" OR "25 hydroxyvitamin d" OR calcidiol OR calcifediol OR cholecalciferol OR ergocalciferol OR "25-hydroxycholecalciferol" OR "25-hydroxyergocalciferol" OR ercalcidiol)
Embase	('medication related osteonecrosis of the jaw'/exp OR 'medication related osteonecrosis of the jaw':ti,ab,kw OR 'jaw osteonecrosis'/exp OR 'jaw osteonecrosis':ti,ab,kw OR mronj:ti,ab,kw OR onj:ti,ab,kw OR bronj:ti,ab,kw OR aronj:ti,ab,kw OR ((medication:ti,ab,kw OR drug*:ti,ab,kw OR bisphosphonate*:ti,ab,kw OR antiresorpt*:ti,ab,kw OR denosumab:ti,ab,kw OR antiangiogen*:ti,ab,kw) AND (osteonecros*:ti,ab,kw OR 'bone necrosis':ti,ab,kw OR necros*:ti,ab,kw) AND (jaw*:ti,ab,kw OR maxillofacial:ti,ab,kw OR mandib*:ti,ab,kw OR maxill*:ti,ab,kw)) OR (osteonecros* NEAR/3 jaw):ti,ab,kw) AND ('vitamin d'/exp OR 'vitamin d deficiency'/exp OR 'cholecalciferol'/exp OR 'ergocalciferol'/exp OR 'calcifediol'/exp OR '25 hydroxyvitamin d'/exp OR 'vitamin d':ti,ab,kw OR 'vitamin d status':ti,ab,kw OR 'hypovitaminosis d':ti,ab,kw OR '25(oh)d':ti,ab,kw OR '25-oh-d':ti,ab,kw OR 25ohd:ti,ab,kw OR '25-hydroxyvitamin d':ti,ab,kw OR calcidiol:ti,ab,kw OR calcifediol:ti,ab,kw OR '25-hydroxycholecalciferol':ti,ab,kw OR '25-hydroxyergocalciferol':ti,ab,kw OR ercalcidiol:ti,ab,kw)

Online Resource 2A. Newcastle-Ottawa Quality Assessment Scale – Cohort study.

Studies	Representativeness of the exposed cohort	Selection of the non-exposed cohort	Ascertainment of exposure	Demonstration that outcome of interest was not present at start of study	Comparability of cohorts on the basis of the design or analysis (study adjusts for age☆, sex☆)	Assessment of outcome	Was follow-up long enough for outcomes to occur	Adequacy of follow up of cohorts	Score (☆)
Park et al. (2022)		☆	☆	☆		☆		☆	5

Online Resource 2B. Newcastle-Ottawa Quality Assessment Scale – Case–control studies.

Studies	Is the case definition adequate?	Representativeness of the case	Selection of Controls	Definition of Controls	Comparability of cases and controls on the basis of the design or analysis (study adjusts for age☆, sex☆)	Ascertainment of exposure	Same method of ascertainment for cases and controls	Non-response rate	Score (☆)
Shirani et al. (2021)				☆		☆	☆		3
Picardo et al. (2020)	☆	☆		☆		☆	☆		5
Heim et al. (2017)	☆			☆		☆	☆		4
Thumbigere-Math et al. (2016)	☆			☆	☆	☆	☆		5
Tsao et al. (2013)	☆			☆	☆☆	☆	☆		6

☆One score of the Newcastle-Ottawa Quality Assessment Scale.
