# Supplementary material for: Serum vitamin D Levels in patients with medication-related osteonecrosis of the jaw: a systematic review and meta-analysis
Source: BMC Oral Health. 2026 Apr 14;26:1040. doi: 10.1186/s12903-026-08303-9 (PMC13270808; doi:10.1186/s12903-026-08303-9)
Supplement: Supplementary file 2 — Supplementary Material 2. [file 12903_2026_8303_MOESM2_ESM.pdf]

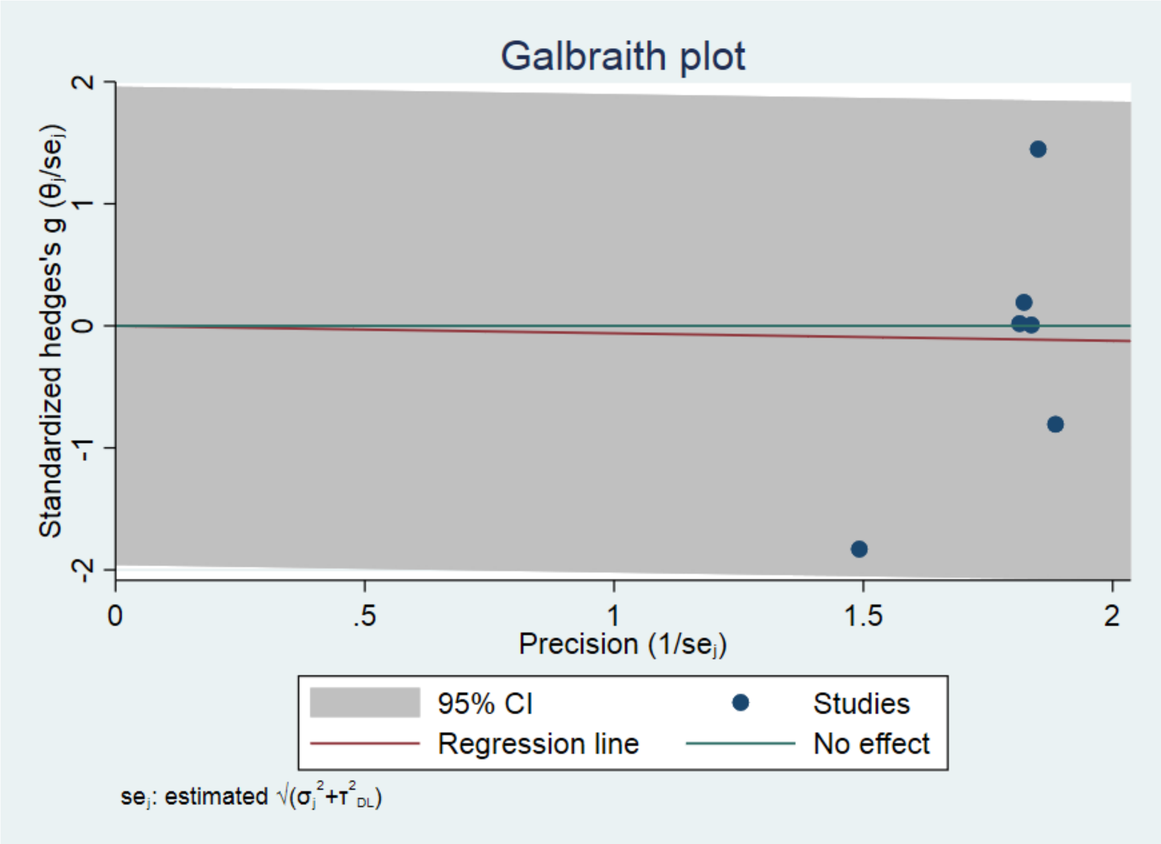

Online Resource 3. Galbraith plot of the standardized mean difference in serum 25(OH) vitamin D levels between MRONJ patients and controls.

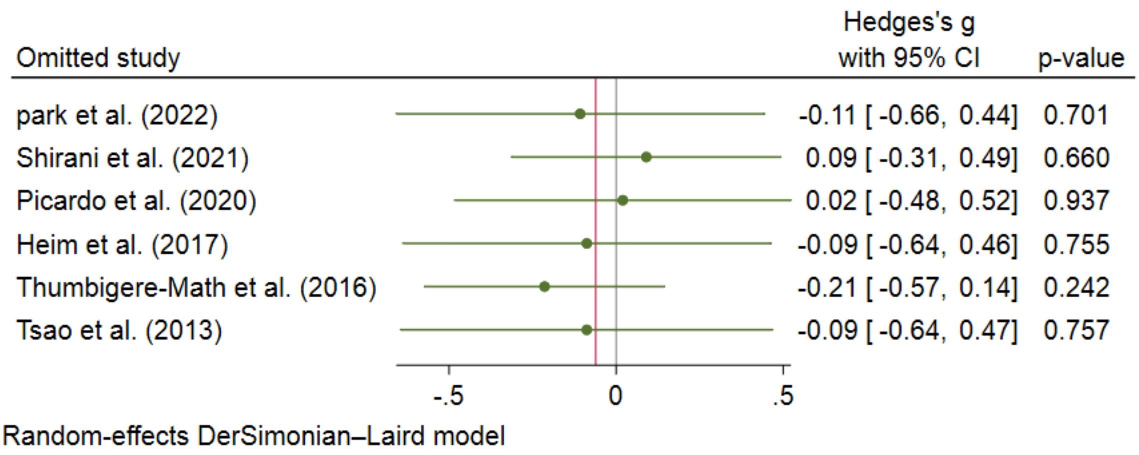

Online Resource 4. Forest plot of sensitivity analysis.

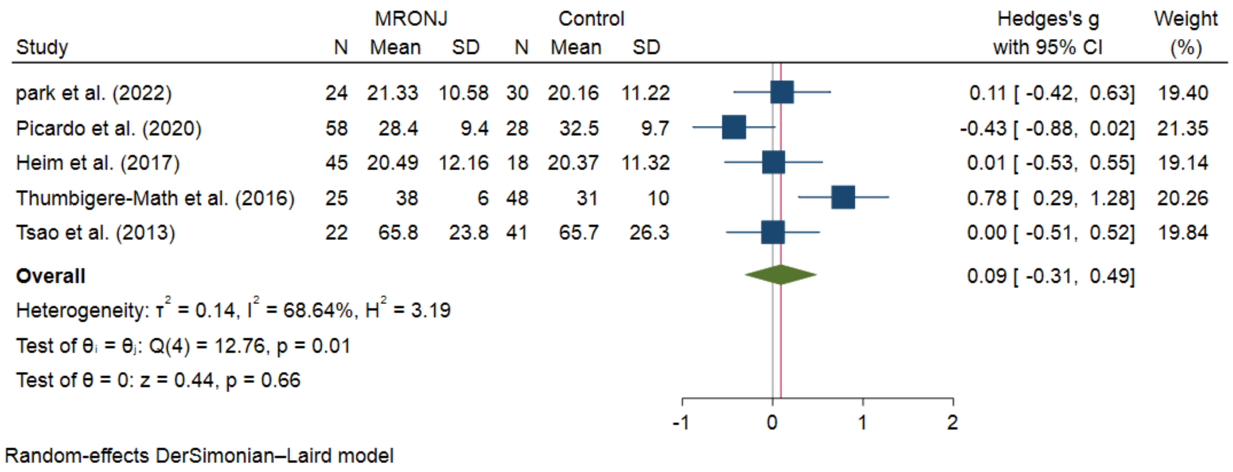

Online Resource 5. Forest plot of the standardized mean difference in serum 25(OH) vitamin D levels between MRONJ patients and controls after excluding the study by Shirani et al.

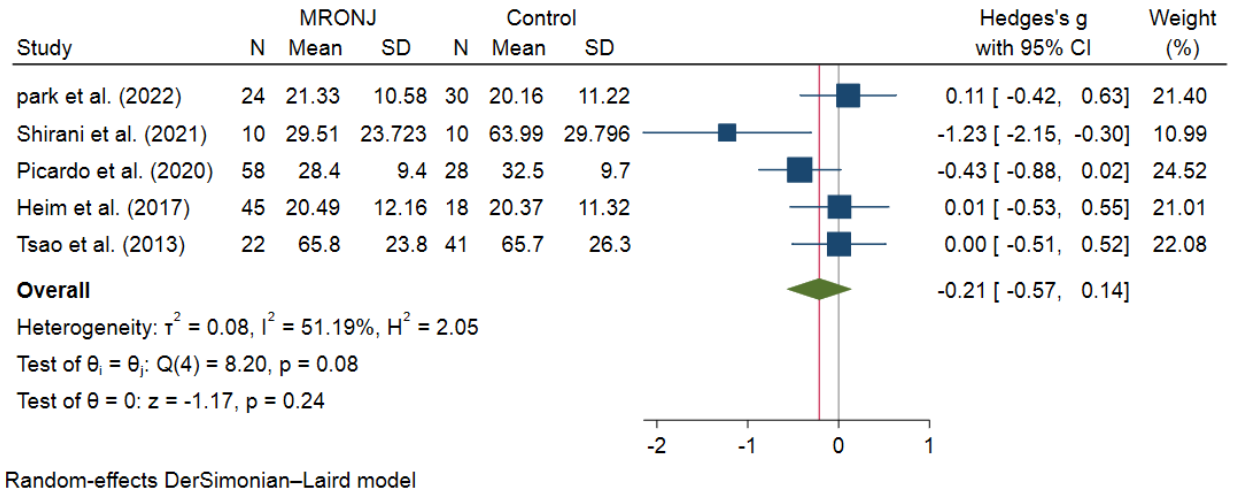

Online Resource 6. Forest plot of the standardized mean difference in serum 25(OH) vitamin D levels between MRONJ patients and controls after excluding the study by Thumbigere-Math et al.

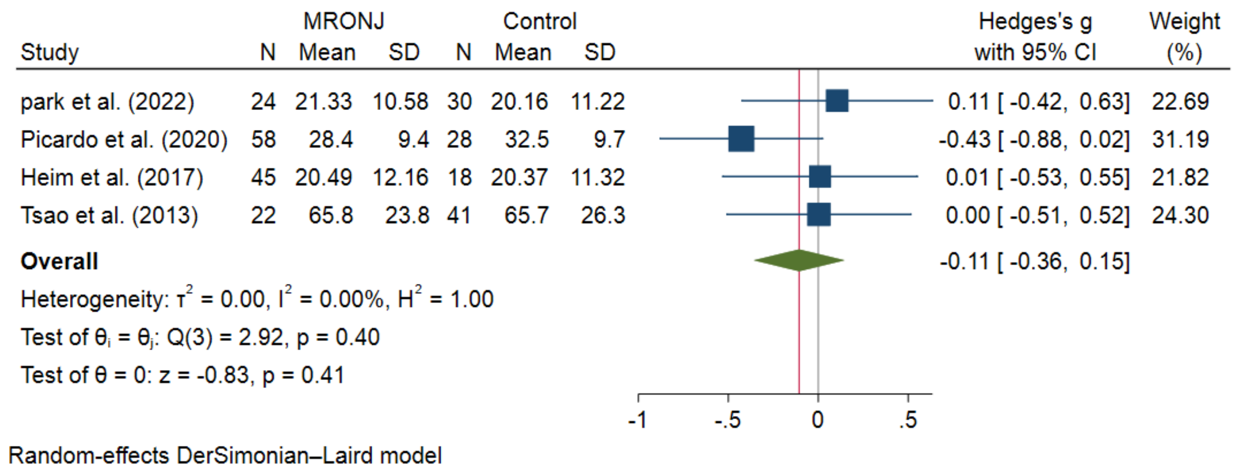

Online Resource 7. Forest plot of the standardized mean difference in serum 25(OH) vitamin D levels between MRONJ patients and controls after excluding the outlier studies.

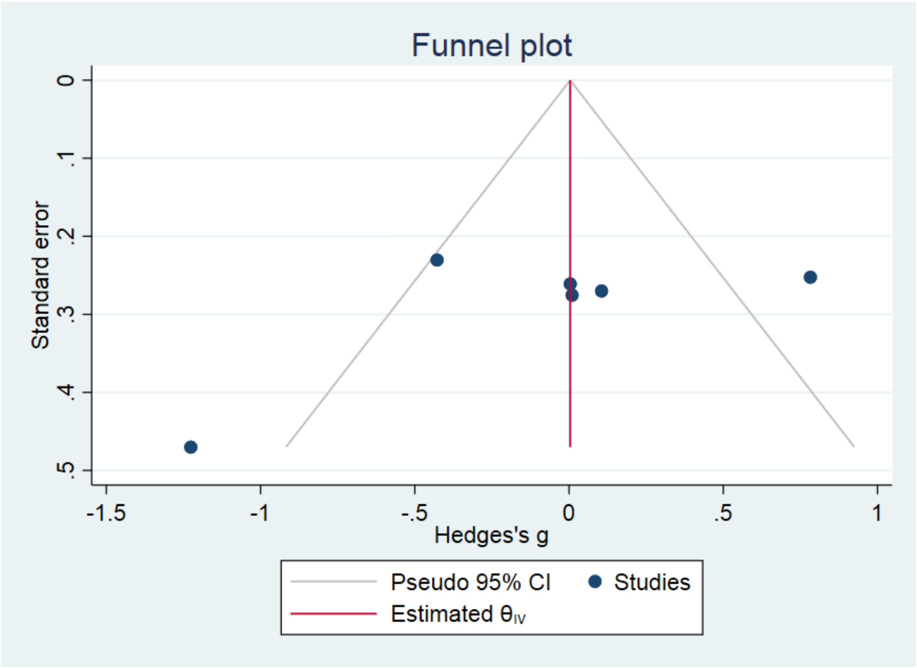

Online Resource 8. Funnel plot of studies. This funnel plot displays slight asymmetry, yet Egger's and Begg's tests reveal no significant publication bias (Egger's P = 0.077, Begg's P = 0.707).
